# Tanshinone IIA accelerates zebrafish venous vascular repair via macrophage recruitment through the CXCR4A–CXCL12B signaling axis

**DOI:** 10.3389/fimmu.2026.1792022

**Published:** 2026-03-19

**Authors:** Li Zhang, Lijuan An, Yanchi Zhao, Zheng Lu, Jiangtao Huang, Lingling Tang, Mengzhu Lv, Dashuang Mo, Liping Shu

**Affiliations:** 1 Center for Tissue Engineering and Stem Cell Research, Translation Medicine Research Center, Guizhou Biomanufacturing Laboratory, Department of Immunology, School of Basic Medicine, Guizhou Medical University, Guiyang, China; 2 National and Guizhou Joint Engineering Laboratory for Cell Engineering and Biomedicine Technique, and Guizhou Province Key Laboratory for Regenerative Medicine, Guizhou Medical University, Guiyang, Guizhou, China

**Keywords:** CXCR4A-CXCL12B, macrophages, tanshinone IIA, vascular repair, zebrafish

## Abstract

**Background:**

Vascular injury is a major contributor to the development of cardiovascular diseases. Following vascular damage, macrophages migrate to the injury site and, during the later stages of vascular repair, secrete cytokines such as interleukin-10 (IL-10) and transforming growth factor-β1a (TGFB1A), thereby promoting vascular regeneration. Previous studies have demonstrated that macrophage recruitment to sites of tissue injury is mediated by the CXCR4A-CXCL12B signaling axis. In a screening of traditional Chinese medicinal herbs for cardiovascular therapeutic potential, *Salvia miltiorrhiza* root was identified as a promising source of bioactive compounds capable of enhancing vascular repair through modulation of the CXCR4A-CXCL12B axis.

**Methods:**

Establishing a vascular injury model in transgenic zebrafish lines *Tg* (*flk1*:eGFP; *gata1*:dsRed) using a two-photon microscopy laser system. Dynamic monitoring of vascular repair via two-photon microscopy. Evaluate macrophage migration capacity in a *Tg* (*mpeg1*:eGFP) zebrafish vascular injury model using confocal microscopy. Detection of *il-10* and *tgfb1a* expression released by macrophages via qPCR experiments. Detect CXCR4A-CXCL12B expression at the site of zebrafish vascular injury via fluorescence *in situ* hybridization coupled with antibody staining.

**Results:**

We confirm that compounds from the selected extract promote macrophage migration to vascular injury sites by upregulating the CXCR4A-CXCR12B signaling axis. This process accelerates repair of damaged blood vessels in zebrafish by inducing the release of cytokines such as *il-10* and *tgfb1a*.

**Conclusions:**

This study confirms that *Salvia miltiorrhiza*, a traditional Chinese medicinal plant, is a valuable source of bioactive compounds with pro-angiogenic properties. Our findings provide scientific support for the traditional use of *Salvia miltiorrhiza* active components in treating vascular injuries.

## 1 Introduction

Cardiovascular diseases (CVDs) occur with increasing frequency worldwide and remain a leading cause of mortality. Vascular dysfunction plays a central role in the development of many CVDs, making the restoration of vascular health a critical therapeutic goal (1). Endothelial cells (ECs) are key regulators of vascular remodeling, maintaining homeostasis by mediating vasodilation, contraction, and overall vascular function (2, 3).

Current pharmacological treatments for CVD include β-blockers, calcium channel blockers, angiotensin-converting enzyme inhibitors, anticoagulants, diuretics, and platelet aggregation inhibitors (4). Although effective, these agents are often limited by serious adverse effects. For example, high-dose β-blockers have been associated with harmful outcomes in high-risk myocardial infarction patients with heart failure or cardiogenic shock (5), and statins can cause complications such as rhabdomyolysis (6). Consequently, traditional Chinese medicine has attracted growing attention as a complementary or alternative therapy, owing to its long history of clinical use and generally fewer side effects (7).

*Salvia miltiorrhiza* Bunge (The plant name originates from http://mpns.kew.org November 10, 2025), a perennial herb of the Labiatae family (8), exhibits broad biological activities, including antibacterial, antioxidant, and anti-inflammatory effects. It is widely used to treat hyperlipidemia, stroke, and both cardiovascular and cerebrovascular diseases (9). Among its active constituents, Tanshinone IIA (TAN IIA)—a fat-soluble diterpene—shows particularly potent cardiovascular and cerebrovascular protective effects, including inhibition of atherosclerosis (AS) (10). TAN IIA reduces oxidative stress and inflammatory damage in ECs improves endothelial dysfunction, and inhibits proliferation and migration of vascular smooth-muscle cells (VSMCs), thereby limiting vascular stenosis (11). It has also been reported to attenuate atherosclerosis through macrophage-mediated pathways (12). Despite these findings, the pharmacological mechanisms of TAN IIA remain incompletely understood, and its role in vascular repair is largely unexplored.

Macrophages are essential mediators of vascular regeneration. Their remarkable plasticity allows them to adapt to diverse microenvironments and adopt distinct functional phenotypes. In response to specific stimuli, macrophages polarize toward either the pro-inflammatory M1 or the anti-inflammatory, pro-repair M2 phenotype through complex signaling and transcriptional networks. Macrophages coordinate angiogenesis, inflammation, and extracellular matrix remodeling during all stages of tissue repair, from the initial inflammatory response to resolution and remodeling (13–16). The transition from M1 to M2 macrophages is particularly important for wound healing and tissue regeneration (17). M2 macrophages, induced by cytokines such as IL-4 and IL-10 (18–21), secrete high levels of anti-inflammatory mediators including IL-10 and TGF-β, thereby suppressing inflammation and promoting repair (22–29). They also facilitate extracellular matrix synthesis and remodeling—producing fibronectin, collagen, and proteoglycans—to support tissue regeneration (30–32). In addition, M2 macrophages efficiently clear apoptotic cells and debris, helping to resolve inflammation and restore tissue homeostasis (33–36). At later stages, they regulate metabolic processes, promote angiogenesis, and further remodel the extracellular matrix (37–42). Remarkably, M2 macrophages can even differentiate into endothelial-like cells and contribute directly to neovascularization, a process mediated by transcription factors such as Prox1 (43, 44). Consistent with these roles, macrophages have been shown to mediate vascular repair in zebrafish models (45).

The present study aimed to establish a zebrafish vascular-injury model to evaluate the effects of TAN IIA on vascular repair. Our results demonstrate that TAN IIA significantly accelerates the healing of injured vessels. Mechanistically, TAN IIA enhances macrophage recruitment to injury sites and promotes their conversion to M2 macrophages via the CXCR4A-CXCR12B signaling axis, thereby facilitating vascular repair in zebrafish.

## 2 Materials and methods

### 2.1 Zebrafish

Use transgenic zebrafish lines *Tg* (*flk1*:eGFP; *gata1*:dsRed), *Tg* (*flk1*:eGFP), and *Tg* (*mpeg1*:eGFP) at 72 hpf (hours post-fertilization). These lines were bred in-house. The transgenic zebrafish strains *Tg* (*flk1*: eGFP; *gata1*: dsRed), *Tg* (*flk1*: eGFP), and *Tg* (*mpeg1*: eGFP) were maintained and reared under standard laboratory conditions at 26 ± 2 °C. The transgenic adult zebrafish and zebrafish embryos used in this experiment were randomly selected. The study complies with the Animals management regulations (Order No. 2 of the State Science and Technology Commission of the People’s Republic of China, 1988). Permit No. 2403704.

### 2.2 Main reagents

Drug tanshinone IIA (Glpbio, USA, purity >99.50%), carboxymethyl cellulose sodium (Coolaber, China), DMEM basal medium (Gibco, USA), AMD3100 (MCE, USA), SDF-1β (MCE, USA). JAK1 Protein Antibody (UpingBio, CHINA), p-JAK1 Protein Antibody (Servicebio, CHINA), STAT6 Protein Antibody (UpingBio, CHINA), p-STAT6 Protein Antibody (UpingBio, CHINA).

### 2.3 Establishment of a zebrafish vascular injury model

Establish a venous vascular injury model in transgenic zebrafish lines *Tg* (*flk1*: eGFP; *gata1*: dsRed), *Tg* (*flk1*: eGFP), and *Tg* (*mpeg1*: eGFP) by a two-photon laser scanning fluorescence microscopy system. First, zebrafish embryos at 72 hpf were fixed with carboxymethyl cellulose sodium in a Cell Culture Dish (NEST, China) and placed on a microscope stage. A two-photon laser scanning fluorescence system was activated to observe the expression of *Tg* (*flk1*:eGFP; *gata1*:dsRed), *Tg* (*flk1*:eGFP), and *Tg* (*mpeg1*:eGFP). Select the tail vein terminal portion of the zebrafish (length 110 μm) as the laser focus site and use a 920 nm infrared laser to irradiate the focus site for 2 seconds to establish a transgenic zebrafish vein injury model.

### 2.4 Fluorescence *in situ* hybridization coupled antibody staining

Detection of *cxcl12b* and *cxcr4a* gene expression in damaged blood vessels through fluorescence *in situ* hybridization coupled antibody staining (46).

### 2.5 Cell culture and preparation of conditioned medium

Cells from the human endothelial cell line EA. hy926 and the mouse macrophage cell line RAW264.7 were cultured in DMEM basal medium supplemented with 10% fetal bovine serum (FBS), antibiotics (100 mg/mL streptomycin and 100 U/mL penicillin). Both cell lines were cultured at 37 °C in a humidified incubator containing 5% CO_2_. EA. hy926 cells were seeded into 6-well plates (1.5 × 10^4^ cells/well). When the cell density reached 80%, the cells were treated with 1 μg/mL LPS (Sigma, USA) for 24 hours, subsequently treated with TAN IIA (0.5, 1, and 2 μM), and finally cultured in DMEM basal medium supplemented with 0.5% FBS for 24 hours. Then, collect the CM from each group and centrifuge at 2000×g for 10 minutes to collect the supernatant, which is stored at -80 °C for further experiments.

### 2.6 Transwell migration assay

A total of 200 μL of RAW264.7 cell suspension (containing 0.5% FBS DMEM medium) was seeded into the upper chamber of an 8 μm pore size transwell chamber (Corning, USA) at a density of 1 × 10^5^ cells/chamber. The cells were co-cultured with 600 μL of conditioned medium (CM), AMD3100 (10 μM) and SDF-1β (10 μM) from different groups in the lower chamber for 24 hours. Residual macrophages that did not migrate into the upper chamber were removed using a sterile cotton swab. Migrated cells were fixed with 4% PFA for 20 minutes and stained with 0.1% crystal violet (Solarbio, China) for 15 minutes. Finally, migrated macrophages were counted using ImageJ software.

### 2.7 Wound healing

RAW264.7 cells (4 × 10^5^ cells/well) were seeded in a 6-well culture plate (Corning-Costar, USA) for 24 hours to reach 80% confluence, followed by serum starvation for 12 hours. A wound line was created using a 200 μL micropipette tip. CM, AMD3100 (10 μM) and SDF-1β (10 μM) were then added to the 6-well plate, and cells were cultured for 24 h. The wound area was visualized under a microscope and quantified using ImageJ software.

### 2.8 Molecular docking

Obtain cxcl12b (Q6V9B5), cxcr4a (Q7ZU02) and IL 4 (D1YSM1) from the RCSB Protein Data Bank (https://www.rcsb.org) and download AlphaFold (https://alphafold.ebi.ac.uk/). The experimental ligand TAN IIA was sourced from the PubChem database (https://pubchem.ncbi.nlm.nih.gov/) and converted to pdbqt format using OpenBabel software. After processing the molecular structures using PyMOL 3.1.6 and AutoDockTools 1.5.6, the most favorable free energy binding conformation was selected based on the principles described in AutoDock Vina (http://vina.scripps.edu). Visualization was performed using Discovery Studio 2025 Client.

### 2.9 Network pharmacology analysis

Retrieve known targets and SMILES molecular structures for TAN IIA from the DGIdb (https://dgidb.org/), TCMSP (https://www.tcmsp-e.com/), and PubChem (https://pubchem.ncbi.nlm.nih.gov/) databases. Subsequently, the SMILES number was imported into the SwissTargetPrediction (http://www.swisstargetprediction.ch/) online platform to screen for potential targets based on a probability threshold greater than zero. Finally, all target sources were integrated and deduplicated, yielding a total of 100 potential targets for TAN IIA. Identified 3,737 unique targets associated with vascular injury from the Genecards (www.genecards.org), MalaCards (https://www.malacards.org/), and OMIM (https://www.omim.org/) databases. The 100 potential targets for TAN IIA identified in the study were subjected to an intersection analysis with 3,737 targets associated with vascular injury. By plotting a Venn diagram, a total of 60 core targets co-present in both datasets were identified. Import the above intersecting targets into the STRING database (https://cn.string-db.org/), set the confidence threshold to 0.4, and exclude free-floating targets to obtain the protein interaction network.

### 2.10 Quantitative real-time polymerase chain reaction analysis

Total RNA was extracted from transgenic zebrafish and EA. hy926 cells using TRIzol (Thermo, USA), and cDNA was obtained from 1000 ng of total RNA using a reverse transcription kit (Thermo, USA). Then, q-PCR was performed using SYBR Green qPCR pre-mix (Thermo, USA). Relative gene expression was calculated using the 2-ΔΔCT method, with β-actin as the standardized reference. The primers used in this study are listed in **Supplementary Table 1**.

### 2.11 Western blot

After laser-damaging the blood vessels of 3dpf *Tg* (*flk1*:eGFP;*gata1*:dsRed) larvae, proteins were extracted from whole fish using a protein extraction kit (NE-PER™ Nucleus and Cytoplasmic Extraction Reagent, Thermo Scientific). The extracted proteins were subjected to SDS-PAGE electrophoresis following concentration measurement, then transferred to a PVDF membrane.

### 2.12 Statistics

All data are expressed as mean ± standard error of the mean (SEM). Statistical analysis of two groups of samples was performed using Student’s t-test with GraphPad Prism 9 software (GraphPad Software, USA). Statistical analysis of more than two groups of samples was performed using one-way analysis of variance (ANOVA). In all cases, *P* < 0.05 was considered statistically significant.

## 3 Results

### 3.1 Construction of a zebrafish venous vessel injury model

**Figure 1:**
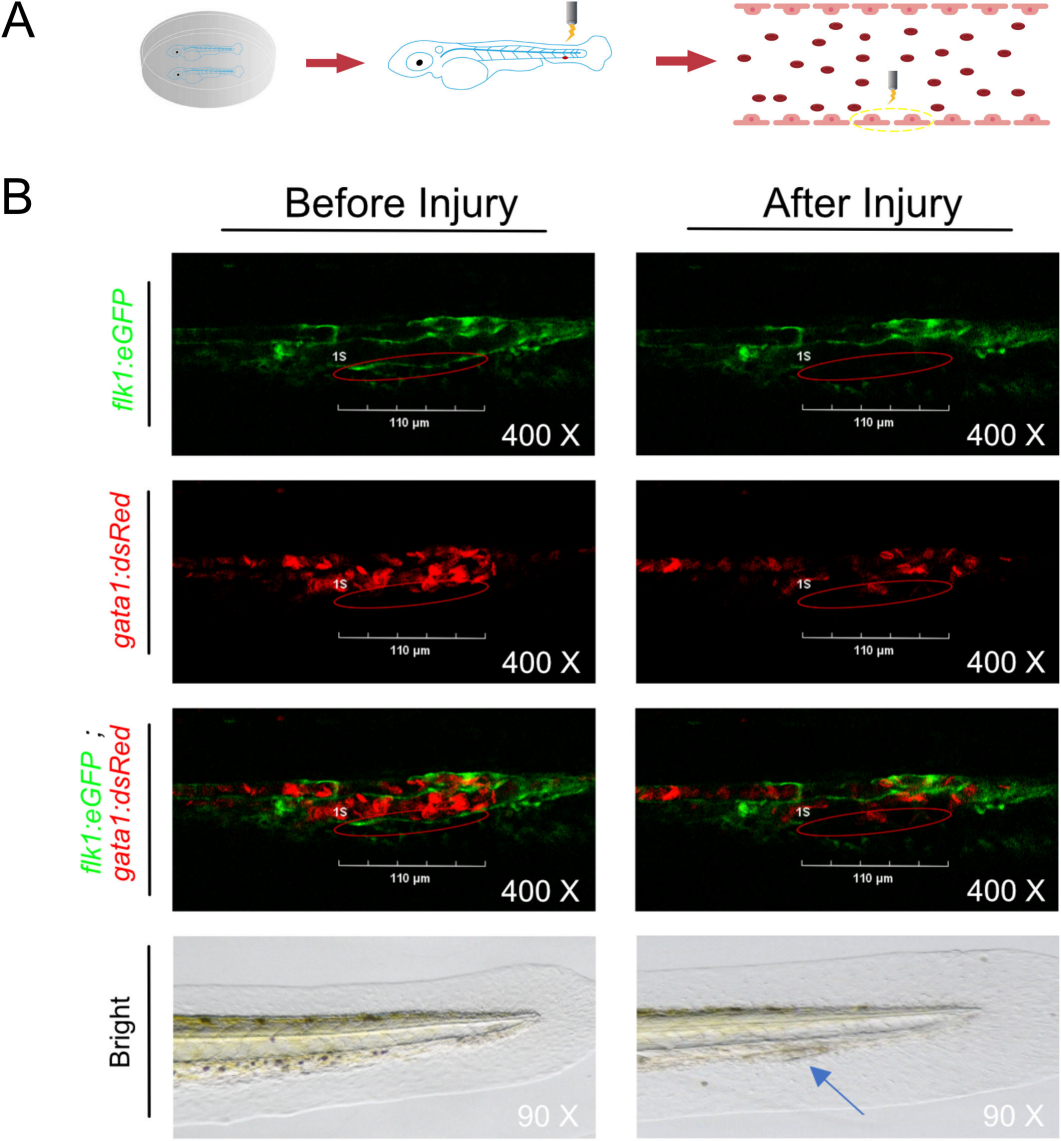
Zebrafish vascular injury model. **(A)** Schematic diagram of establishing a venous vascular injury model using zebrafish embryos (72 hpf). **(B)** The vascular structure and red blood cells can be clearly observed under a two-photon microscope. The tail vein returning to the heart of the zebrafish is marked with an infrared laser system, and the marked vessels are damaged at specific points using infrared laser to construct a zebrafish vascular damage model. Green marks the vessels, red marks the red blood cells, and red ellipses mark the vessels that need to be damaged. The blue arrow indicates the area of vascular damage under white light.

To establish a venous vascular injury model, zebrafish embryos (72 hpf) from the transgenic line *Tg* (*flk1*:eGFP;*gata1*:dsRed) were immobilized in culture dishes with carboxymethyl cellulose. Vessel structures were visualized using two-photon laser scanning fluorescence microscopy. Target vessels were identified and marked, then injured with the microscope’s infrared laser system (**Figure 1A**). Successful vessel damage was confirmed a localized loss of eGFP fluorescence (**Figure 1B**; **Supplementary Video 1**) and by visible surface lesions under bright-field microscopy. These observations confirmed the successful establishment of a zebrafish venous vessel injury model.

### 3.2 TAN IIA promotes vascular repair

**Figure 2:**
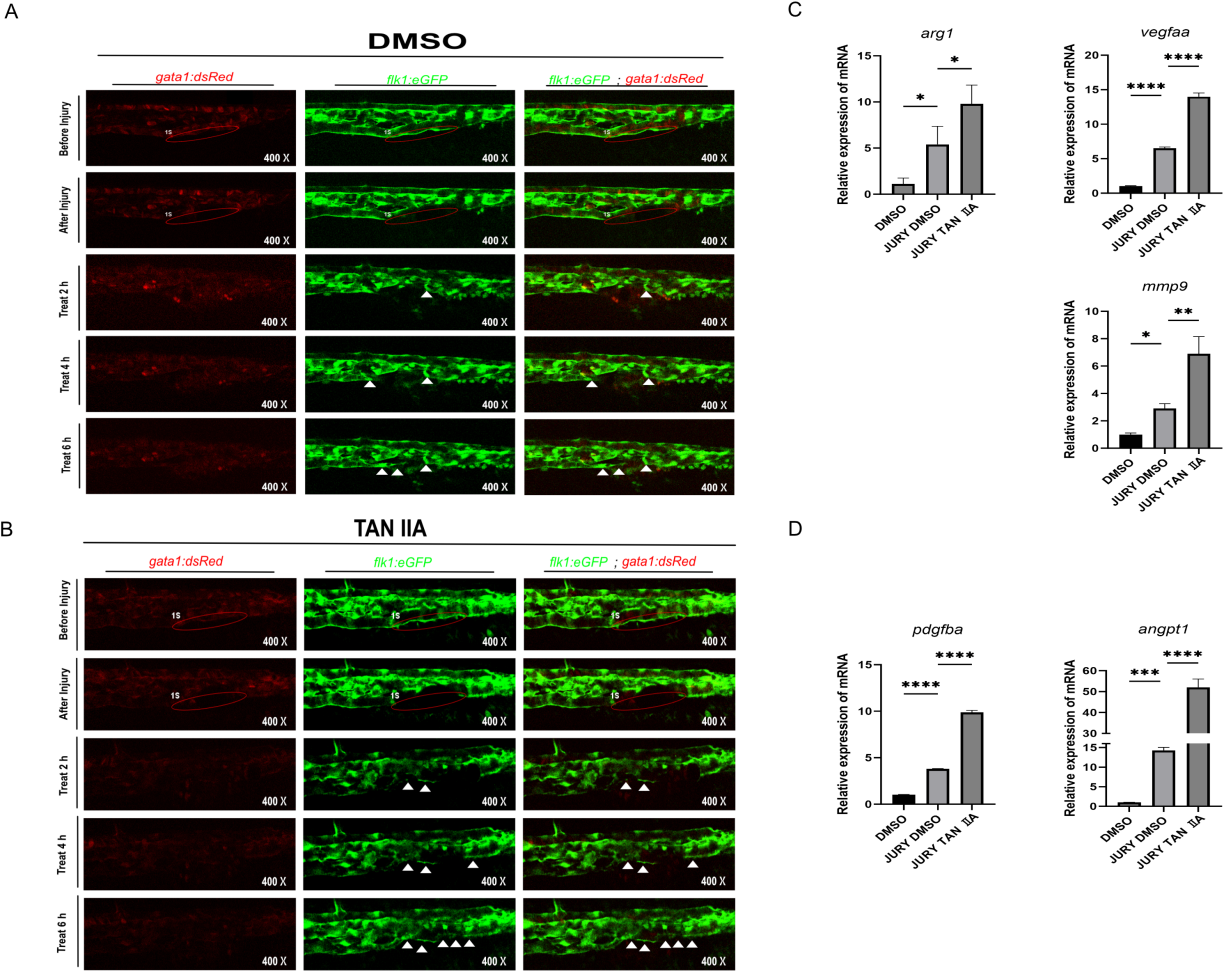
TAN IIA promotes rapid repair of damaged blood vessels in zebrafish. **(A, B)**TAN IIA accelerates the repair of damaged blood vessels in zebrafish. The white triangular arrows indicate the repaired blood vessels. **(C, D)** The effect of TAN IIA (2 μM) and dmso (2 μM) on the mRNA expression levels of *mmp9*, *vegfaa*, *arg1, pdgfba*, and *angpt1* genes. DMSO group refers to zebrafish that did not undergo blood vessels damage. * *P* < 0.1 versus control group, ** *P* < 0.01 versus control group, *** *P* < 0.001 versus control group, **** *P* < 0.0001 versus control group.

Using this model, we examined the effects of TAN IIA—the major bioactive component of *Salvia miltiorrhiza* Bunge—on vascular repair. Injured zebrafish were treated with TAN IIA, and vascular recovery was assessed at 2, 4, and 6 hours post-injury, with DMSO serving as vehicle control. By 4 hours, TAN IIA (2 μM)-treated fish exhibited markedly faster vascular regeneration than controls (47); by 6 hours, damaged vessels were largely restored, whereas DMSO-treated vessels remained incompletely repaired (**Figures 2A, B**). q-PCR analysis revealed that vascular remodeling-related gene (*mmp9*, *vegfaa*, *pdgfba*, *arg1*, *angpt1*) were significantly upregulated in the TAN IIA treatment for 6 hours after vascular injury in zebrafish (**Figures 2C, D**). Collectively, these results indicate that TAN IIA accelerates the vascular repair process in zebrafish.

### 3.3 TAN IIA enhances macrophage recruitment in zebrafish

**Figure 3:**
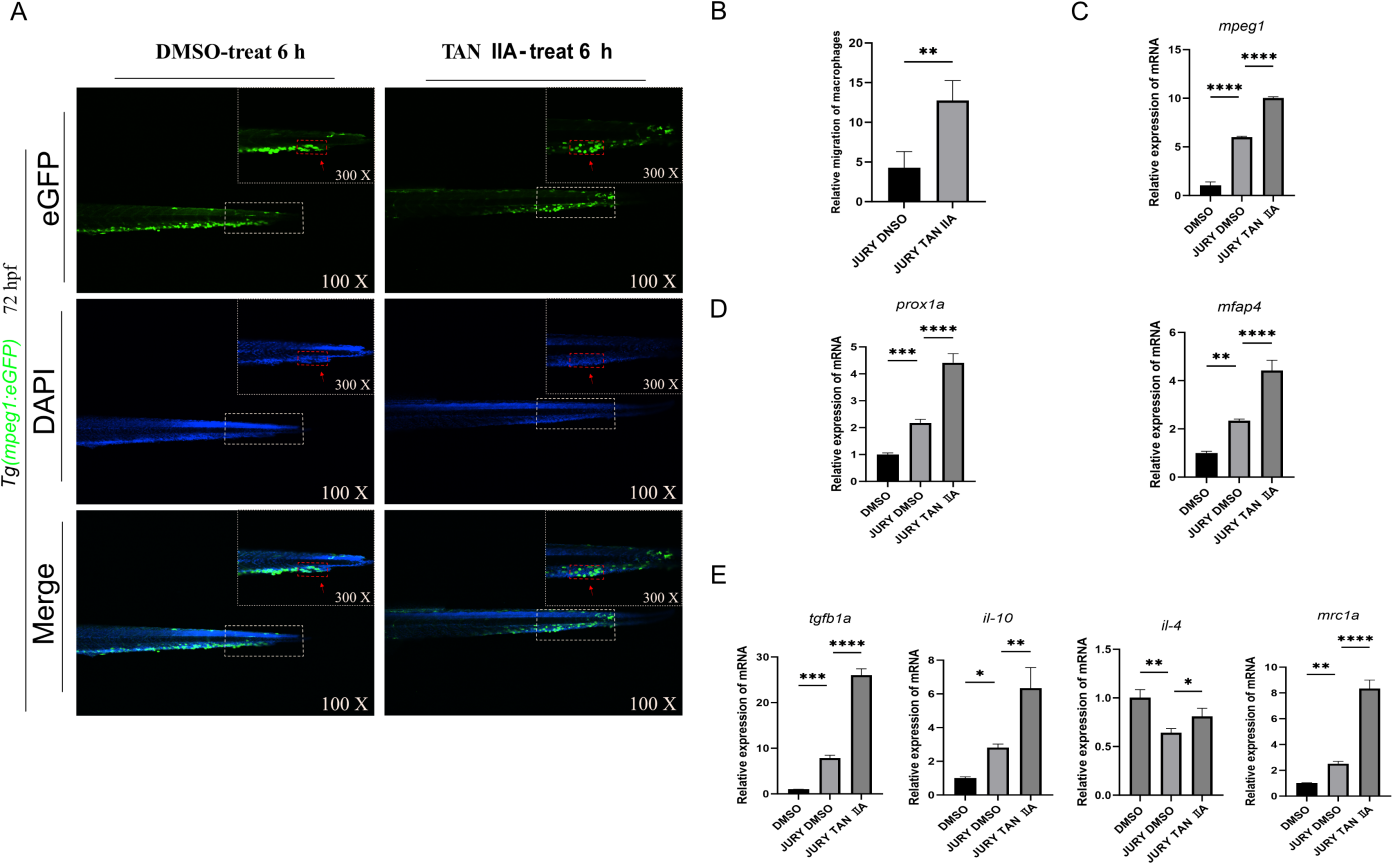
TAN IIA enhances macrophage recruitment to sites of vascular injury. **(A)** TAN IIA promotes the recruitment of macrophages to sites of vascular injury, green-labeled macrophages. **(B)** Count the number of macrophages within the vascular injury area. **(C)** Effect of TAN IIA on the mRNA expression levels of *mpeg1*. **(D)** Effect of TAN IIA on the mRNA expression levels of *mfap4*, *prox1a*. **(E)** Effect of TAN IIA on mRNA expression levels in M2 macrophages of *mrc1a*, *il-4, tgfb1a, il-10*. The relative mRNA expression of candidate genes (as indicated) was assessed by q-PCR after TAN IIA (2 μM) and dmso (2 μM) treatment for 6h (n = 3, three independent experiments). At 6h, the transcriptional level of *mpeg1, mfap4, prox1a, mcr1a*, *il 4, tgfb1a, il 10* were increased. * *P* < 0.1 versus control group, ** *P* < 0.01 versus control group, *** *P* < 0.001 versus control group, **** *P* < 0.0001 versus control group.

To explain the mechanism by which TAN IIA promotes rapid repair of damaged blood vessels, we focused on macrophages. Previous studies have shown that TAN IIA can slow the progression of cardiovascular disease by modulating macrophage activity, and macrophages, particularly M2 macrophages, are known to participate in vascular and tissue repair (17). To determine whether TAN IIA acts on macrophages during vascular repair, we employed *Tg* (*mpeg1*: eGFP) transgenic zebrafish subjected to vascular injury (**Figure 1**). At 6 hours, TAN IIA-treated larvae displayed a significantly greater number of macrophages recruited to the injury site compared with DMSO-treated controls (**Figures 3A, B**). Moreover, qPCR analysis revealed that TAN IIA treatment for 6 hours significantly up-regulated multiple macrophage and M2 related genes, including general macrophage markers (*mpeg1* and *mfap4*), the M2 surface markers (*mrc1a)*, the polarization factor (*il-4*), anti-inflammatory cytokines (*il-10* and *tgfb1a*), and the pro-angiogenic transcription factor (*prox1a*) (**Figures 3C–E**). These data indicate that TAN IIA promotes macrophage recruitment and polarization toward the M2 phenotype, thereby facilitating vascular repair in zebrafish.

### 3.4 Identifying potential targets for TAN IIA and vascular injury through network pharmacology analysis

**Figure 4:**
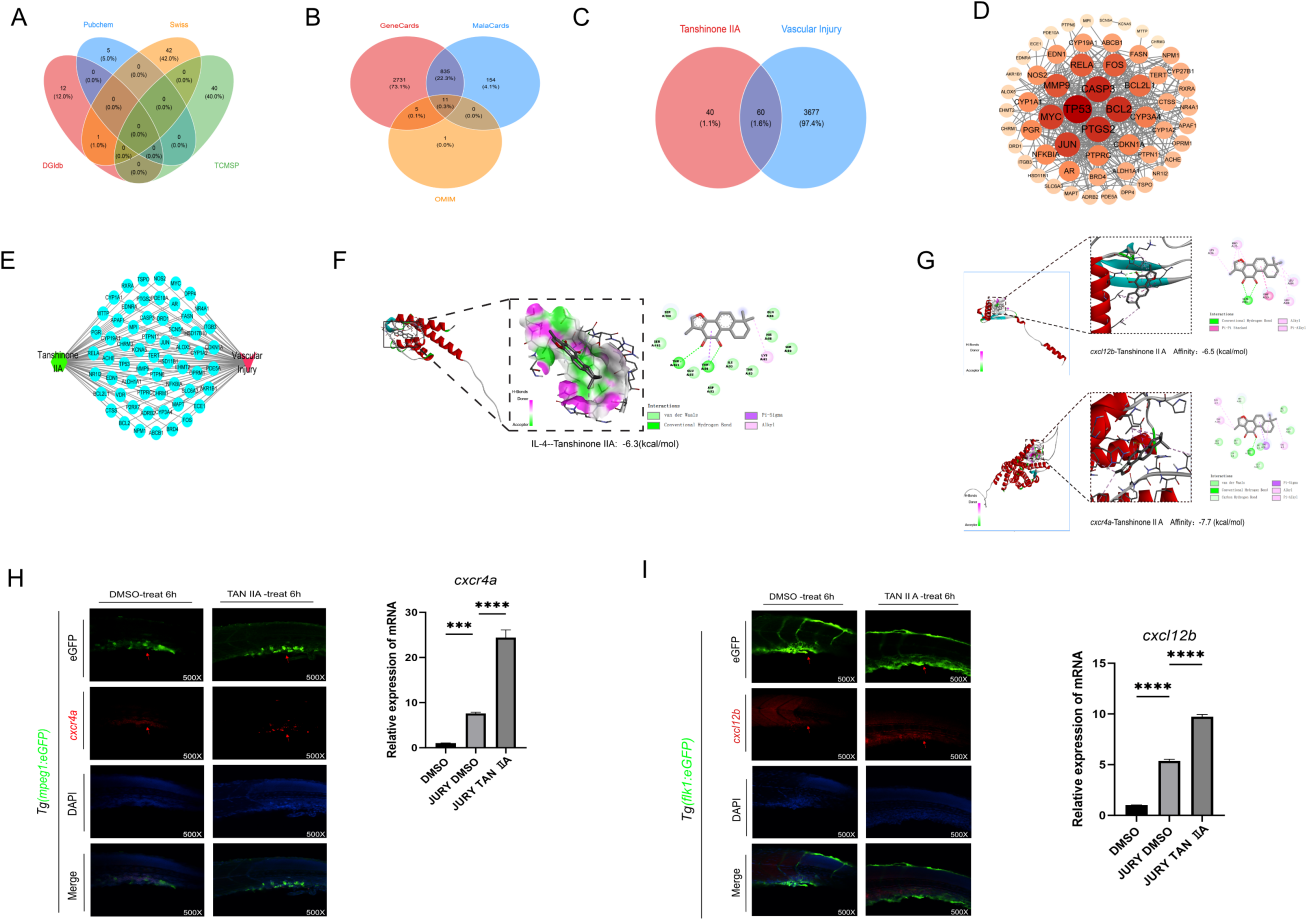
TAN IIA may promote macrophage recruitment by activating the CXCR4A-CXCL12B signaling axis. **(A)** Potential Targets for TAN IIA. **(B)** Unique Targets Associated with Vascular Injury. **(C)** TAN IIA acts on the core target of vascular injury. **(D)** Construction of the Protein-Protein Interaction Network for TAN IIA and Vascular Injury Proteins. **(E)** TAN IIA and Vascular Injury Drug-Target-Disease Network Diagram. **(F, G)** Molecular Docking of Tanshinone IIA with IL-4, CXCR4A and CXCL12B. **(H)** TAN IIA promotes macrophage aggregation in damaged blood vessels and enhances *cxcr4a* expression at sites of vascular injury. Red arrows indicate sites of vascular injury. Effects of TAN IIA (2 μM) and DMSO (2 μM) on *cxcr4a* mRNA expression levels. **(I)** TAN IIA promotes repair of damaged blood vessels and induces high expression of *cxcl12b* at sites of vascular injury. Red arrows indicate sites of vascular injury. Effects of TAN IIA (2 μM) and DMSO (2 μM) on *cxcl12b* mRNA expression levels. The relative mRNA expression of candidate genes (as indicated) was assessed by q-PCR after TAN IIA (2 μM) and dmso (2 μM) treatment for 6h (n = 3, three independent experiments). ***P < 0.001 versus control group. ****P < 0.0001 versus control group.

Previous studies have demonstrated that the CXCR4A-CXCL12B signaling axis plays a crucial role in restoring homeostasis in damaged tissues and blood vessels (48). CXCL12, secreted by endothelial cells, is known to promote macrophage migration through activation of CXCR4 (49–51). To further elucidate the role of TAN IIA in vascular injury, we identified relevant targets linking TAN IIA to vascular injury diseases through network pharmacology analysis. We identified MMP9, a core functional gene of M2 macrophages, as a central target in the TAN IIA-vascular injury disease network(**Figures 4A–E**). Molecular docking results indicate that TAN IIA exhibits strong binding affinity for cxcr4a, cxcl12b, and IL-4, suggesting potential regulatory interactions between TAN IIA and these molecules (**Figures 4F, G**). We therefore hypothesized that TAN IIA may enhance the secretion of CXCL12B by vascular endothelial cells, thereby activating *cxcr4a* and facilitating macrophage migration toward sites of vascular injury. To test this hypothesis, we used *Tg* (*flk1*: eGFP) and *Tg* (*mpeg1*: eGFP) transgenic zebrafish lines and assessed *cxcl12b* expression in endothelial cells and *cxcr4a* expression in macrophages at the injury site by fluorescence *in situ* hybridization combined with antibody staining. TAN IIA treatment group exhibited higher expressions of the *cxcl12b* and *cxcr4a* at the injury site compared with DMSO group. These findings indicate that TAN IIA enhances *cxcl12b* expression in endothelial cells, thereby promoting the recruitment of *cxcr4a*-expressing macrophages to injured vessels. In addition, qPCR analysis confirmed consistent up-regulation of these genes at the mRNA level (**Figures 4H, I**). Collectively, these data demonstrate that TAN IIA may promote macrophage recruitment by activating the CXCR4A-CXCL12B signaling axis.

### 3.5 TAN IIA promotes macrophage migration *in vitro*

**Figure 5:**
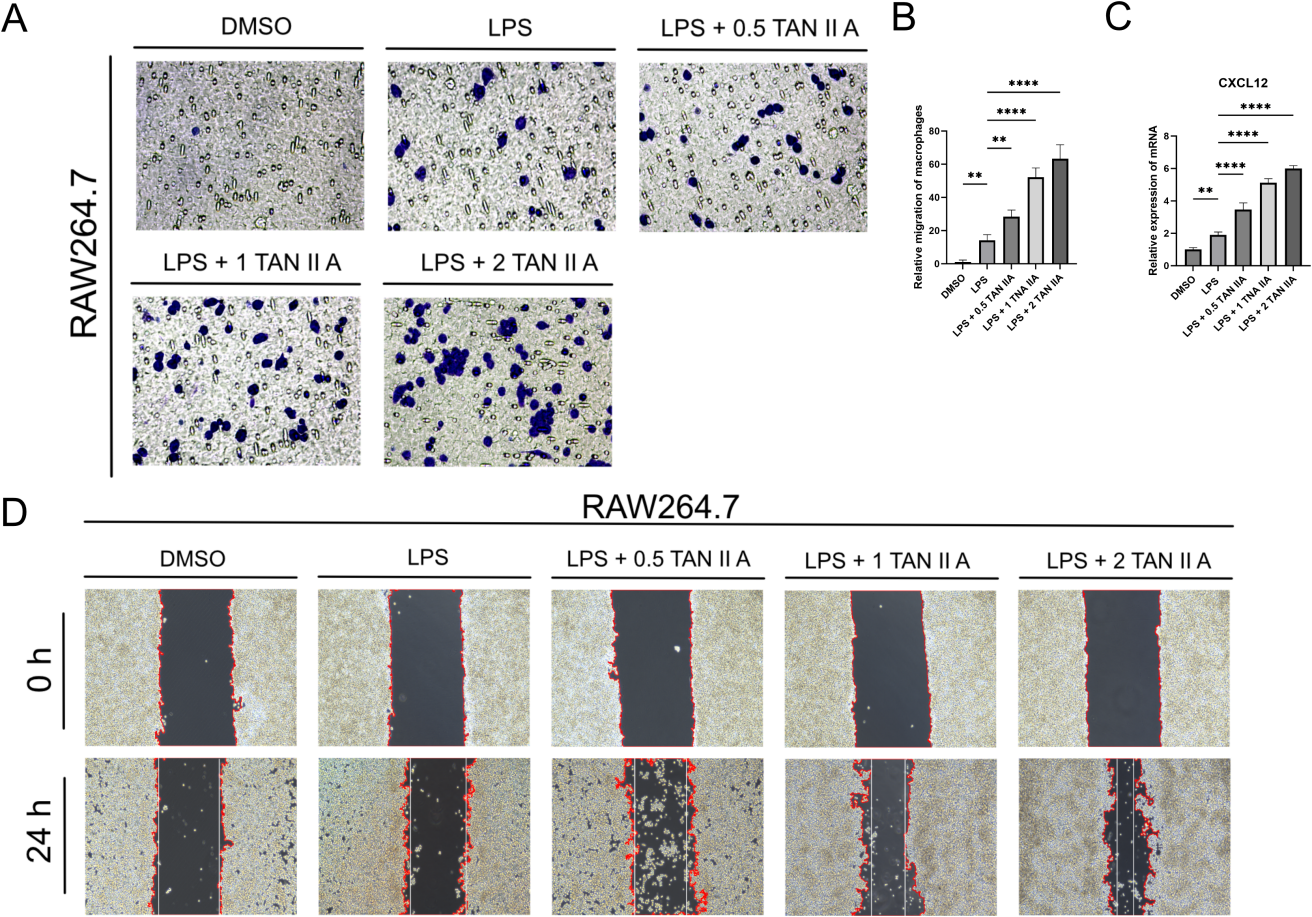
TAN IIA-treated CM promotes migration of RAW264.7 macrophages. **(A)** CM obtained from EA. hy926 cells damaged by LPS (1 μg/L) and treated with TAN IIA significantly induced RAW264.7 cell migration. The migration of RAW264.7 cells was analyzed using the Transwell assay, and cells were photographed after 24 hours of treatment with increasing concentrations of LPS (1 μg/L) and TAN IIA (0.5, 1, and 2 μM) (n = 5). **(B)** Compared with the DMSO control group and the LPS-damaged group, TAN IIA treatment increased the number of migrating cells. Quantitative analysis of the above cell Transwell assay. **(C)** Effect of TAN IIA on CXCL12 mRNA expression levels in EA. hy926 cells. The relative mRNA expression levels of CXCL12 were determined by q-PCR in EAhy.926 cells treated with LPS (1 μg/L) for 24 hours and then treated with TAN IIA (0.5, 1, and 2 μM) for 24 hours (n = 3, three independent experiments). After 24 hours, the CXCL12 expression level increased in the TAN IIA -treated group. **(D)** RAW264.7 cells wound healing experiment: After uniformly calculating the cell wound edges using ImageJ software, the white vertical lines indicate the degree of wound healing. ** *P* < 0.01 versus control group, **** *P* < 0.0001 compared to the control group.

To further validate these findings *in vitro*, we used human umbilical vein endothelial cells (EA.hy926) and murine macrophages (RAW264.7). EA.hy926 cells were first stimulated with LPS (1 µg/L) to mimic vascular inflammation, followed by treatment with increasing concentrations of TAN IIA (0.5, 1, or 2 µM). The conditioned medium (CM) from TAN IIA–treated endothelial cells and applied to Transwell migration assays. TAN IIA-treated CM significantly increased RAW264.7 migration compared with both DMSO and LPS-only controls (**Figures 5A, B**). Consistently, a wound-healing assay produced similar results (**Figure 5D**). Furthermore, q-PCR showed that TAN IIA up-regulated LPS-induced CXCL12 expression in EA.hy926 cells (**Figure 5C**). These findings indicate that TAN IIA stimulates endothelial cells to secrete CXCL12, thereby promoting macrophage migration.

### 3.6 TAN IIA promotes repair of damaged blood vessels in zebrafish via the CXCR4A-CXCL12B signaling axis

**Figure 6:**
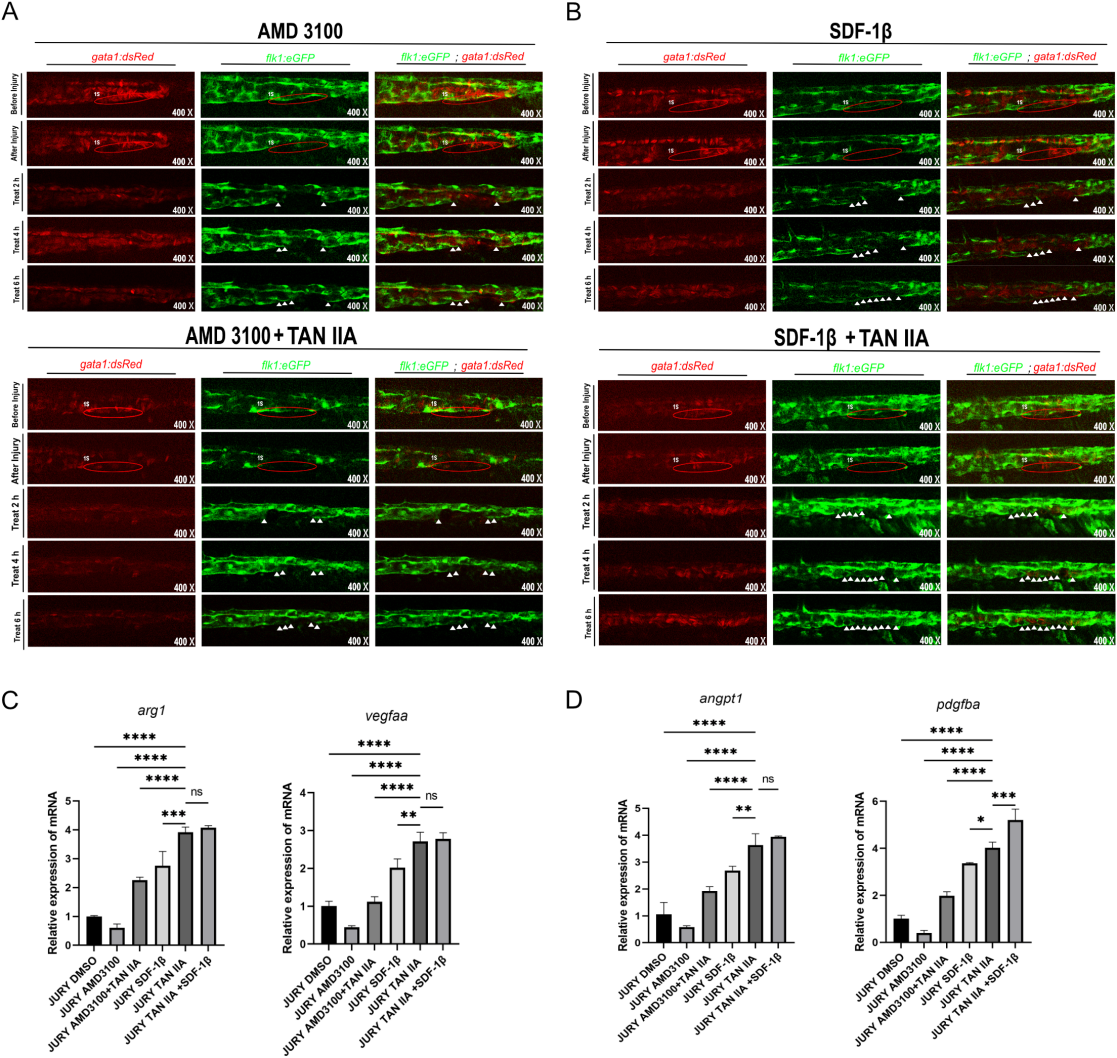
TAN IIA accelerates vascular repair through the CXCR4A-CXCL12B signaling axis. **(A)** AMD3100(10 μM) inhibits the repair of damaged blood vessels in zebrafish, while TAN IIA(2 μM) can rescue this phenomenon. **(B)** SDF-1β(10 μM) can accelerate the repair of damaged blood vessels in zebrafish to a certain extent. The white triangular arrow indicates the repaired blood vessel. **(C, D)** The effect of AMD3100 (10 μM), AMD3100 (10 μM)+TAN IIA (2 μM), SDF-1β(10 μM) and SDF-1β(10 μM)+TAN IIA (2 μM) on the mRNA expression levels of *vegfaa*, *arg1*, *pdgfba*, and *angpt1* genes. * *P* < 0.1 versus control group, ** *P* < 0.01 versus control group, *** *P* < 0.001 versus control group, **** *P* < 0.0001 versus control group.

AMD3100 is an antagonist of CXCR4A that inhibits the CXCR4A-CXCL12B signaling axis (52). SDF-1β is a matrix-derived CXC chemokine that signals through the CXCR4A receptor and upregulates the CXCR4A-CXCL12B signaling axis. In a zebrafish venous injury model, we observed slower vascular repair in the AMD3100 group, while the AMD3100+TAN IIA group accelerated repair compared to the AMD3100 group. Both the SDF-1β group and the SDF-1β+TAN IIA group accelerated vascular repair in zebrafish. Furthermore, the SDF-1β+TAN IIA group resulted in more complete and rapid vascular repair(**Figures 6A, B**). q-PCR analysis revealed that vascular remodeling-related genes (*mmp9*, *vegfaa*, *pdgfba*, *arg1*, *angpt1*) were significantly downregulated in the AMD3100 treatment for 6h, whereas TAN IIA treatment induced their upregulation(**Figures 6C, D**). These findings indicate that the CXCR4A-CXCL12B signaling axis plays a crucial role in zebrafish vascular repair, and TAN IIA can activate this signaling pathway.

### 3.7 TAN IIA promotes M2 macrophage migration through the CXCR4A-CXCL12B signaling axis

**Figure 7:**
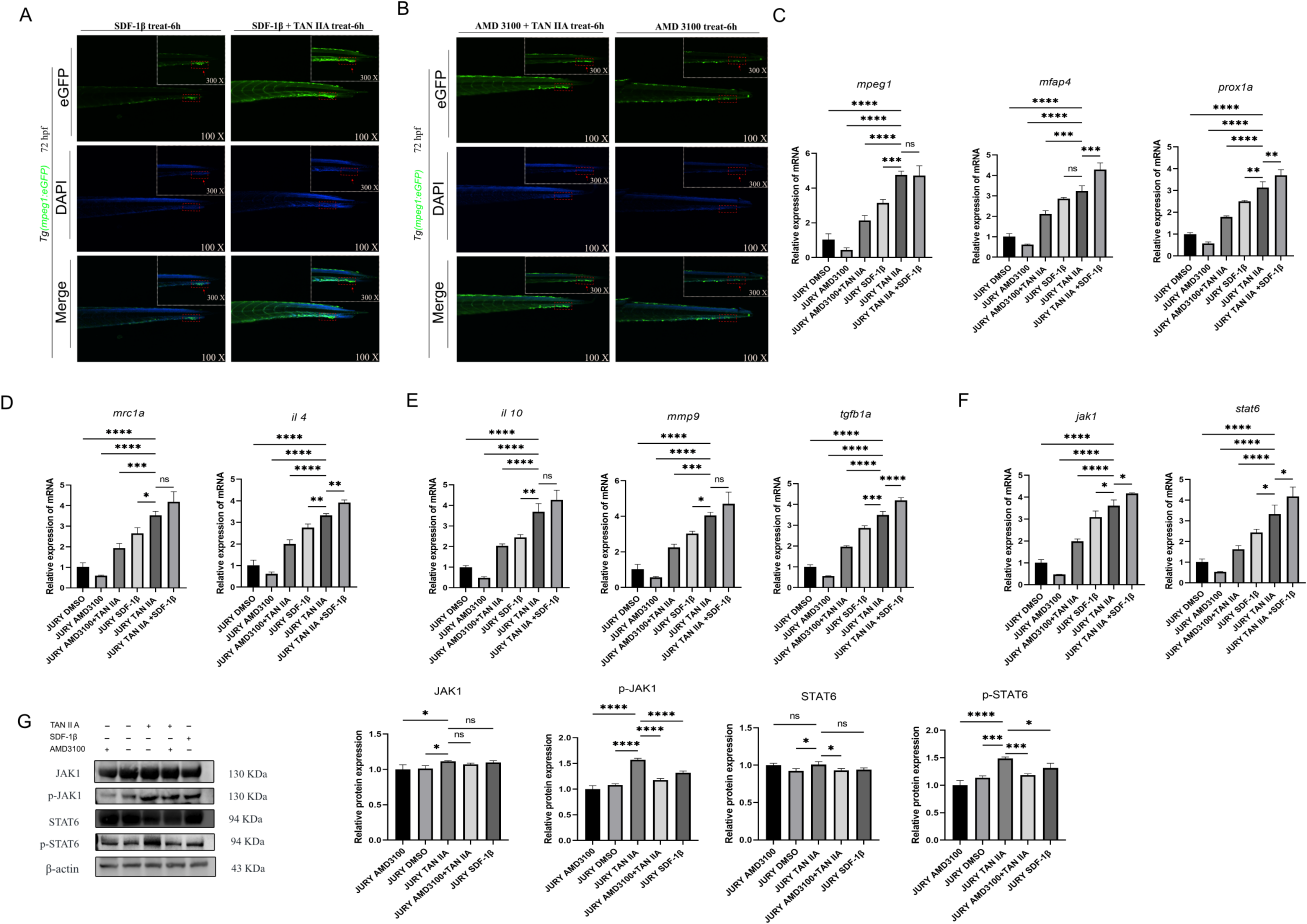
TAN IIA accelerates the recruitment of M2 macrophages through the CXCR4A-CXCL12B signaling axis. **(A)** SDF-1β also promotes macrophage recruitment to sites of vascular injury (green-labeled macrophages) to a certain extent. **(B)** AMD3100 (10 μM) reduces macrophage recruitment to sites of vascular injury, while TAN IIA(2 μM) can rescue this phenomenon. **(C)** Effects of AMD3100 (10 μM), AMD3100 (10 μM)+TAN IIA (2 μM), SDF-1β(10 μM) and SDF-1β(10 μM)+TAN IIA (2 μM) on *mpeg1, mfap4* and *prox1a* mRNA expression levels. **(D, E)** Effects of AMD3100 (10 μM), AMD3100 (10 μM)+TAN IIA (2 μM), SDF-1β(10 μM) and SDF-1β(10 μM)+TAN IIA (2 μM) on *mrc1a, il 4, il 10, mmp9* and *tgfb1a* mRNA expression levels. **(F)** Effects of AMD3100 (10 μM), AMD3100 (10 μM)+TAN IIA (2 μM), SDF-1β(10 μM) and SDF-1β(10 μM)+TAN IIA (2 μM) on *jak1* and *stat6* mRNA expression levels. **(G)** Effects of AMD3100 (10 μM), AMD3100 (10 μM)+TAN IIA (2 μM), SDF-1β(10 μM) on the expression levels of Jak1 and Stat6 proteins. Relative mRNA expression levels of candidate genes (as shown in the figure) were measured by q-PCR 6 hours after treatment with AMD3100 (10 μM), AMD3100 (10 μM)+TAN IIA (2 μM), SDF-1β(10 μM) and SDF-1β(10 μM)+TAN IIA (2 μM) (n=3, three independent experiments). *P < 0.1 versus control group, **P < 0.01 versus control group, ***P < 0.001 versus control group, ****P < 0.0001 versus control group.

In the previous experimental results, we observed that AMD3100 inhibits the rate of vascular repair in zebrafish. To elucidate this phenomenon, we treated zebrafish with AMD3100, SDF-1β, and TAN IIA after inducing venous vascular injury. The AMD3100 group exhibited reduced macrophage migration at the vascular injury site, while the AMD3100+TAN IIA group showed greater macrophage migration than the AMD3100 group. The SDF-1β+TAN IIA group demonstrated higher macrophage migration than the AMD3100 group(**Figures 7A, B**). q-PCR analysis revealed that macrophage-associated genes (*mpeg1*, *mfap4* and *prox1a*) and M2macrophage-associated genes(*mrc1a*, *mmp9*, *il 4*, *il 10*, *tgfb1a*)were significantly downregulated in the AMD3100 group, whereas TAN IIA treatment induced their upregulation(**Figures 7C–E**). Additionally, Western blotting and q-PCR experiments demonstrated that TAN IIA promotes the upregulation of the JAK1/STAT6 pathway (**Figures 7F, G**). These findings indicate that TAN IIA promotes macrophage migration to sites of vascular injury to accelerate vascular repair in zebrafish via the CXCR4A-CXCL12B signaling axis.

### 3.8 TAN IIA promotes macrophage migration through the CXCR4A-CXCL12B signaling axis *in vitro*

**Figure 8:**
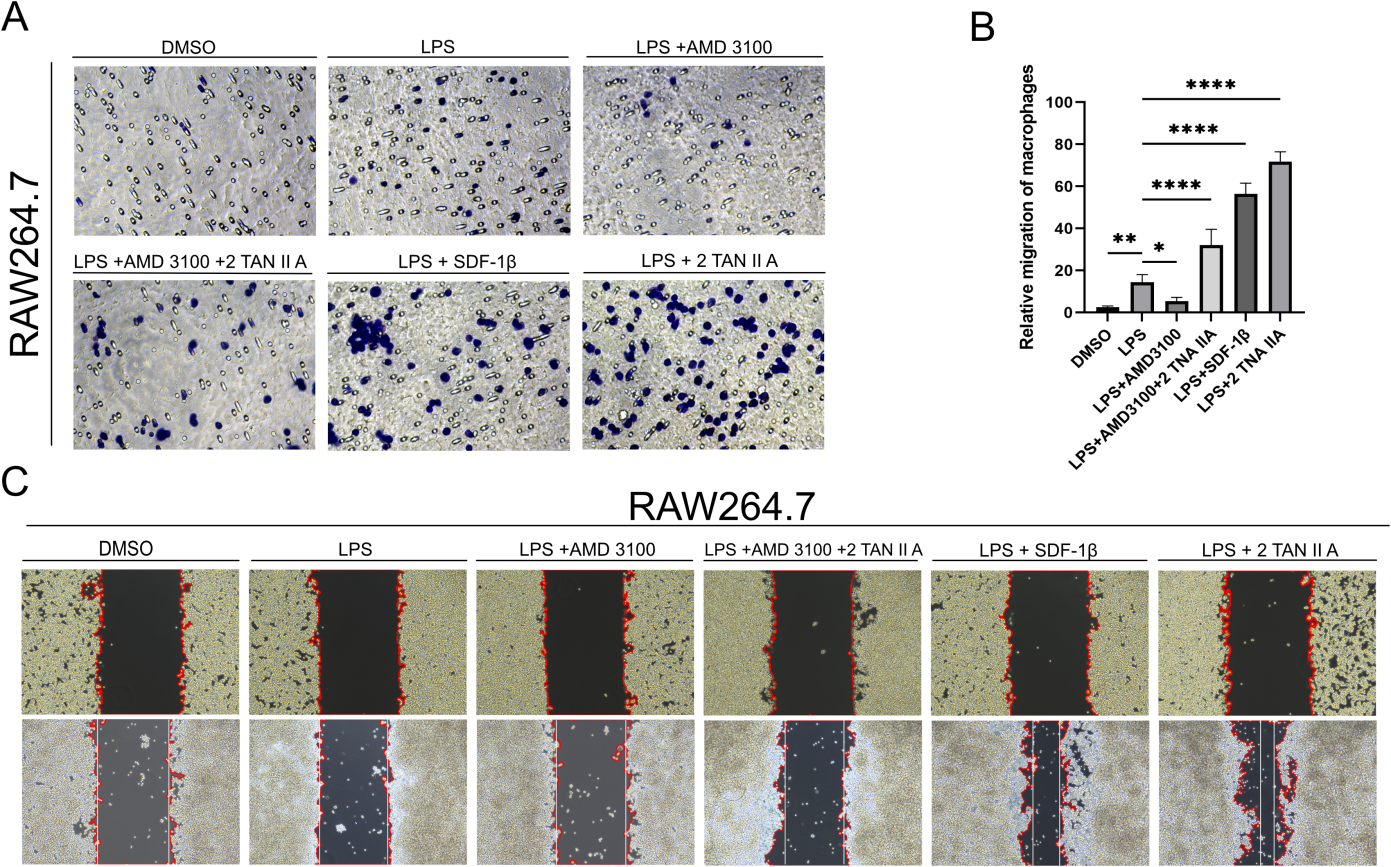
TAN IIA promotes macrophage migration through the CXCR4A-CXCL12B signaling axis. **(A)** Conditioned medium (CM) obtained from EA. hy926 cells injured by LPS (1 μg/L) significantly inhibited the migration of RAW264.7 cells after 24-hour treatment with AMD3100. Transwell assays analyzed RAW264.7 cell migration, with images captured after 24-hour treatment in different CM groups (n=5). Compared to the DMSO control and LPS-injured groups, AMD3100 treatment significantly suppressed the number of migrating cells, while TAN IIA could rescue this effect. **(B)** Quantitative analysis of the cell Transwell assay. **(C)** Wound healing assay in RAW264.7 cells: After uniformly calculating the cell wound edges using ImageJ software, the white vertical line indicates the extent of wound healing. * *P* < 0.1 versus control group, ** *P* < 0.01 versus control group, ****P < 0.0001 versus control group.

To validate the role of AMD3100 in *in vitro* murine macrophages (RAW264.7) migration, we demonstrated through Transwell migration assays that AMD3100 inhibited macrophage migration, and TAN IIA could rescue this effect (**Figures 8A, B**). Wound healing assays confirmed identical results(**Figure 8C**). These findings confirm that TAN IIA can rescue the inhibition of macrophage migration induced by AMD3100.

### 3.9 TAN IIA promotes vascular repair in zebrafish via the CXCR4A-CXCL12B signaling axis

**Figure 9:**
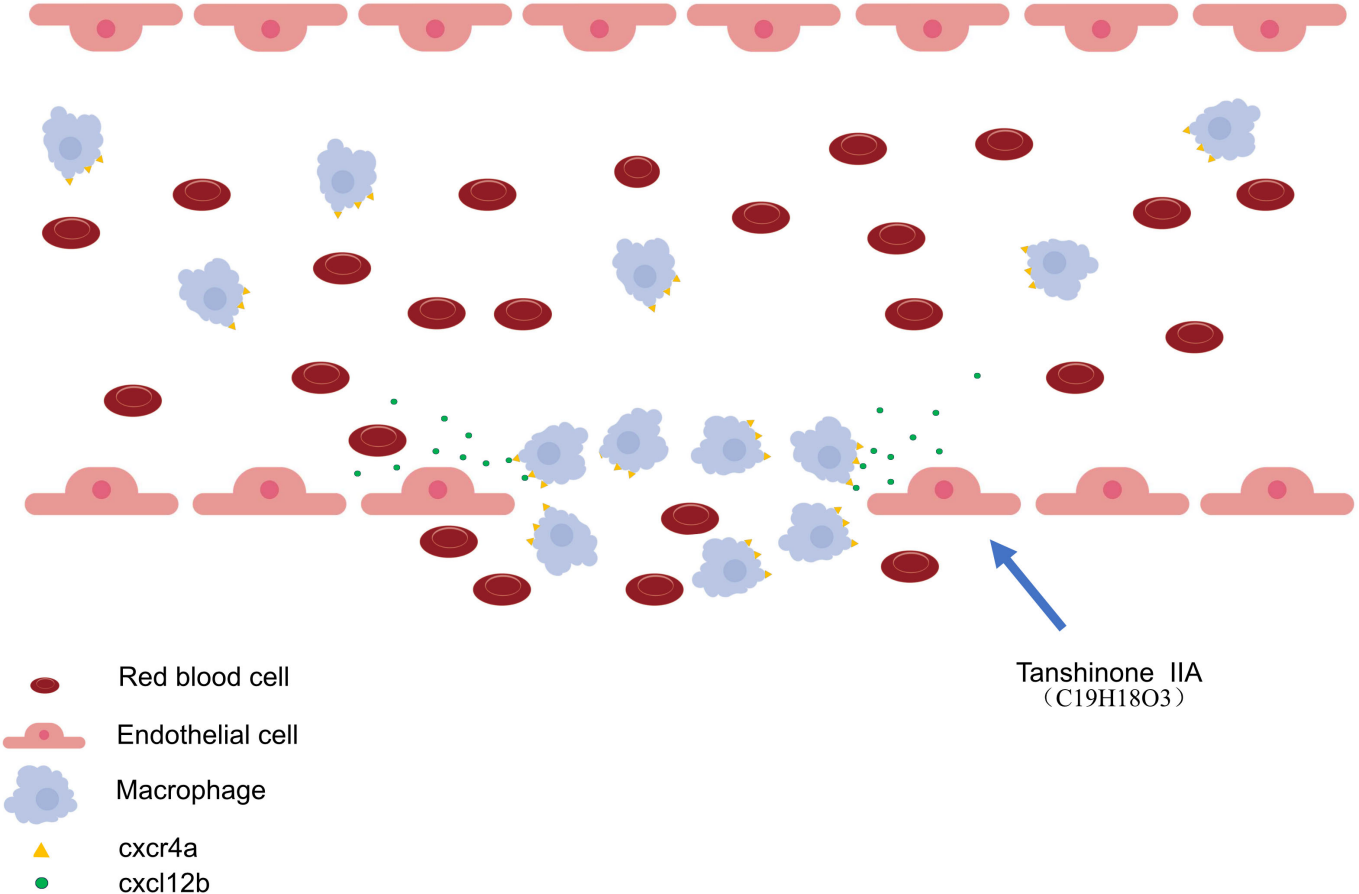
Mechanism diagram of TAN IIA promoting repair of damaged blood vessels. TAN IIA treatment enhances chemokine CXCL12B secretion by endothelial cells, thereby increasing macrophage recruitment via activation of the CXCR4A-CXCL12B signaling axis and ultimately facilitating the repair of damaged blood vessels in zebrafish.

Two-photon infrared laser–induced vessel injury experiments (**Figure 9**) show that TAN IIA stimulates endothelial cells to secrete *cxcl12b*, which activates *cxcr4a* in macrophages. Activation of this signaling pathway promotes macrophage recruitment and M2 polarization, accelerating the repair of injured blood vessels in zebrafish.

## 4 Discussion

*Salvia miltiorrhiza* Bunge, a perennial plant of the Labiatae family (8), possesses diverse biological activities, including antioxidant, antibacterial, and anti-inflammatory effects. Owing to these properties, it has been widely used in the treatment of cardiovascular and cerebrovascular diseases (9). TAN IIA, a fat-soluble diterpene quinone and the principal bioactive component of *S. miltiorrhiza*, exhibits multiple cardiovascular and cerebrovascular benefits. It can inhibit oxidative stress and inflammatory injury in vascular endothelial cells, thereby improving endothelial dysfunction, and protects vascular smooth muscle cells by suppressing their proliferation and migration, which reduces vascular stenosis (11). However, its therapeutic effects in the context of vascular injury remain incompletely characterized, and its potential role in vascular repair has not been fully explored.

In this study, we demonstrate for the first time that TAN IIA promotes vascular repair in a laser-induced zebrafish vein injury model. Its mechanism of action involves enhancing macrophage recruitment by activating the CXCR4A-CXCL12B signaling axis and accelerating vascular repair through M2 macrophages.

Cardiovascular disease has become a major global health challenge, and vascular integrity is a critical determinant of disease onset and progression. Pathological changes in blood vessels are central to many cardiovascular disorders. Previous work has shown that TAN IIA can help prevent vascular dysfunction (11). Building on these findings, our results reveal that TAN IIA markedly enhances repair of infrared-laser-induced venous injury in zebrafish. Moreover, the up-regulation of vascular remodeling genes such as *arg1, mmp9*, *vegfaa*, *pdgfba*, and *angpt1* (51, 53–56) suggests that TAN IIA promotes vascular regeneration at the molecular level.

Macrophages are multifunctional immune cells that play pivotal roles in tissue and vascular remodeling (14–16). Recent studies have highlighted their importance in promoting vascular regeneration (13). Consistent with these findings, our results show that TAN IIA induces macrophage accumulation at sites of vascular injury and up-regulates macrophage-related genes. *In vitro*, conditioned medium from TAN IIA–treated EA.hy926 endothelial cells enhanced RAW264.7 macrophage migration, accompanied by increased CXCL12 expression, indicating that endothelial-derived CXCL12 mediates TAN IIA–induced macrophage chemotaxis. M2 macrophages—also referred to as anti-inflammatory macrophages—express MRC1 as a surface marker (57) and can be induced by the immune regulatory cytokine IL-4 (18, 19). These cells produce anti-inflammatory mediators, such as IL-10 and TGF-β, which contribute to vascular repair and homeostasis (22, 23, 27, 29). Additional markers of macrophage identity and function include *mpeg1* and *mfap4*, which are also involved in hematopoiesis and myeloid cell development (58, 59). Notably, *mfap4* promotes vascular smooth-muscle proliferation and neovascularization (60), while prox1a supports late-stage vascular remodeling (43). Our study revealed that TAN IIA significantly upregulates the expression of *il 4*, *mrc1a*, *il 10*, and *tgfb1a* indicating that it promotes vascular repair by enhancing *il 10* and *tgfb1a* secretion through M2 macrophages. Concurrently, TAN IIA also increased the expression of *mfap4* and *prox1a*, further confirming its role in vascular remodeling.

The CXCR4/CXCL12 signaling axis is known to guide macrophage migration toward injured vessels. CXCL12, secreted by endothelial cells, mediates unidirectional migration of mononuclear macrophages through CXCR4 activation (49, 50, 61). Consistent with this pathway, we observed that TAN IIA up-regulated *cxcr4a* and *cxcl12b* expression at the injury site, and enhanced CXCL12 expression in EA.hy926 cells. These findings suggest that TAN IIA promotes macrophage recruitment to damaged vessels via the CXCR4A-CXCL12B axis.

To further validate this finding, we treated zebrafish with vascular injuries using AMD3100 and SDF-1β. AMD3100 is a CXCR4A antagonist (52), while SDF-1β promotes the migration and infiltration of macrophages expressing CXCR4A (62). We found that AMD3100 inhibited macrophage migration to the site of vascular injury in zebrafish and suppressed repair of damaged blood vessels, whereas TAN IIA reversed this effect. Similar results were obtained in *in vitro* experiments using RAW264.7 cells. Furthermore, we observed that SDF-1β promotes macrophage migration to injured zebrafish blood vessels and accelerates vascular repair, while TAN IIA exhibits a stronger angiogenic effect. Network pharmacology analysis revealed that MMP9, a core functional gene of M2 macrophages, plays a crucial role in TAN IIA and vascular injury. Molecular docking experiments revealed strong binding affinity between TAN IIA and IL4, CXCR4A, and CXCL12B. Furthermore, Western blotting and q-PCR experiments demonstrated that TAN IIA promotes upregulation of the JAK1/STAT6 pathway and enhances the expression of M2 macrophage-related genes (*mrc1a*, *mmp9*, *il 4*, *il 10*, *tgfb1a*). We hypothesize that TAN IIA may also promote macrophage conversion to M2 macrophages, though the mechanism remains unexplored. We propose that TAN IIA accelerates the repair of damaged blood vessels in zebrafish via M2-type macrophages.

This study confirms that TAN IIA accelerates the repair process of zebrafish venous vessels by recruiting macrophages through the CXCR4A-CXCL12B signaling axis. It is known that endothelial nitric oxide synthase (eNOS) serves as a key molecular target for TAN IIA ‘s vascular regulatory effects (63). Previous studies have demonstrated that TAN IIA plays a crucial role in the prevention and treatment of cardiovascular diseases by activating the eNOS pathway (64, 65). However, no direct experimental evidence currently exists to confirm a direct link between TAN IIA ‘s regulation of eNOS and the specific process of macrophage recruitment. Based on this, we hypothesize that TAN IIA may indirectly facilitate CXCL12B-mediated macrophage repair of damaged vessels by regulating eNOS activity in vascular endothelial cells, thereby maintaining vascular integrity and stabilizing the local microenvironment. Future experimental validation of the specific role of the eNOS pathway in TAN IIA ‘s regulation of macrophage recruitment will be a key subsequent research direction for elucidating the molecular mechanism by which TAN IIA treats vascular injury through macrophage recruitment.

In summary, our results identify macrophage recruitment as a key driver of rapid vascular repair in zebrafish. TAN IIA accelerates the repair of damaged zebrafish blood vessels by promoting M2 macrophage recruitment through activation of the CXCR4A-CXCL12B signaling pathway. As a widely used cardiovascular agent derived from *Salvia miltiorrhiza* Bunge, TAN IIA shows strong potential as a therapeutic candidate for vascular injury and regeneration.

## Data Availability

The original contributions presented in the study are included in the article/**Supplementary Material**. Further inquiries can be directed to the corresponding authors.
